# The Use of Telehealth Technology to Support Health Coaching for Older Adults: Literature Review

**DOI:** 10.2196/23796

**Published:** 2021-01-29

**Authors:** Carl Markert, Farzan Sasangohar, Bobak J Mortazavi, Sherecce Fields

**Affiliations:** 1 Department of Industrial and Systems Engineering Texas A&M University College Station, TX United States; 2 Center for Outcome Research Houston Methodist Houston, TX United States; 3 Department of Computer Science and Engineering Texas A&M University College Station, TX United States; 4 Department of Psychological and Brain Sciences Texas A&M University College Station, TX United States

**Keywords:** telemedicine, remote sensing technology, health coaching, decision support systems, clinical, older adults

## Abstract

**Background:**

Health coaching is an intervention process for driving behavior change through goal-setting, education, encouragement, and feedback on health-related behaviors. Telehealth systems that include health coaching and remote monitoring are making inroads in managing chronic conditions and may be especially suited for older populations.

**Objective:**

This literature review aimed to investigate the current status of health coaching interventions incorporating telehealth technology and the associated effectiveness of this intervention to deliver health care with an emphasis on older adults (aged 65 and older).

**Methods:**

A literature review was conducted to identify the research conducted on health coaching combined with remote monitoring for delivering health care to older adults. The Ovid MEDLINE and CINAHL databases were queried using a combination of relevant search terms (including middle aged, aged, older adult, elderly, health coaching, and wellness coaching). The search retrieved 196 papers published from January 2010 to September 2019 in English. Following a systematic review process, the titles and abstracts of the papers retrieved were screened for applicability to health coaching for older adults to define a subset for further review. Papers were excluded if the studied population did not include older adults. The full text of the 42 papers in this subset was then reviewed, and 13 papers related to health coaching combined with remote monitoring for older adults were included in this review.

**Results:**

Of the 13 studies reviewed, 10 found coaching supported by telehealth technology to provide effective outcomes. Effectiveness outcomes assessed in the studies included hospital admissions/re-admissions, mortality, hemoglobin A_1c_ (HbA_1c_) level, body weight, blood pressure, physical activity level, fatigue, quality of life, and user acceptance of the coaching program and technology.

**Conclusions:**

Telehealth systems that include health coaching have been implemented in older populations as a viable intervention method for managing chronic conditions with mixed results. Health coaching combined with telehealth may be an effective solution for providing health care to older adults. However, health coaching is predominantly performed by human coaches with limited use of technology to augment or replace the human coach. The opportunity exists to expand health coaching to include automated coaching.

## Introduction

### Overview of Chronic Diseases

Chronic diseases are health-related conditions that require ongoing medical attention or limit one’s daily activities [1]. These conditions are common among older adults and were the leading causes of death among older adults (aged 65 and older) in the United States in 2017 [2]. Chronic disease management within the world’s aging population is creating a burden on the health care industry [3]. For example, the average medical expenditures in the United States within this older population were 2.6 times the national average and accounted for over one-third of medical spending in 2010 [4]. A subsequent survey by the Kaiser Family Foundation found that older adults (age 55 and over) in the United States accounted for 56% of all health care spending in 2016 but made up only 29% of the population [5].

The Census Bureau projects that the US population aged 65 or older will grow from 49 million in 2016 to 95 million by 2060 [6]. Ninety percent of these older adults prefer to *age in place*, or remain in their homes as they grow older [7] which could also mitigate health care costs for this population compared to the cost of assisted living communities. Aging in place allows them to better maintain contact with friends and family, but this preference presents a challenge for determining what health-related technology is needed to help meet this desire [8]. Telehealth may be one way to effectively manage chronic diseases among older adults while also enabling them to live at home, especially with a number of opportunities available to assist aging in place through advancements in smart sensing technology [9]. Furthermore, the COVID-19 pandemic has also shown the necessity of understanding the efficacy of telehealth systems, as these systems may be the only mode of non-emergency health care delivery for vulnerable populations in a pandemic situation [10]. However, despite the increased access to telehealth technologies, implementation strategies that do not address self-management of one’s health care have led to disappointing findings, such as the failure to reduce re-admissions in individuals with heart failure [11,12].

While telehealth has enabled virtual visits with health care professionals, the self-management capabilities of telehealth require special attention to patient engagement and behavior change methods to improve active participation. Health coaching has gained widespread use in the past few years. Two recent systematic reviews found health coaching to be somewhat effective for adults with chronic conditions [13,14]. Kivelä et al [13] found health coaching to be effective for the patient’s physiological, behavioral, and psychological status, specifically, improvements in weight management, physical activity, physical health, and mental health. Oliveira et al [14] found health coaching to be effective in increasing the level of physical activity in older adults but found no significant improvement in quality of life, mobility, or mood. Neither of these studies evaluated health coaching combined with remote monitoring. The goal of our review was to investigate the current status of health coaching interventions that incorporate telehealth remote monitoring technology and the associated effectiveness of this intervention with an emphasis on older adults.

### Background

*Telehealth* is an all-encompassing term for clinical and nonclinical remote health care services and is defined by the Center for Connected Health Policy as “a collection of means or methods for enhancing health care, public health and health education delivery and support using telecommunications technologies” [15]. For the purpose of this literature review, telehealth includes telemedicine, remote patient monitoring (RPM), remote activity monitoring (RAM), decision support systems (DSSs), and health coaching systems.

*Telemedicine* is the use of telecommunication technology to allow health care workers to provide clinical services (eg, medical therapy) to patients remotely [16]. Telemedicine is useful for providing clinical services to patients in sparsely populated areas or places remotely located from a health care facility [17].

*RPM* is the use of electronic devices and telecommunication technology to monitor and transmit patient physiological or metabolic parameters to a digital database that can be accessed by authorized users [18]. RPM usually involves Bluetooth-enabled or internet-connected devices that automatically transmit monitored parameters. RPM can also include electronic wellness questionnaires that elicit information concerning the patient’s well-being and health status.

*RAM* is the use of electronic devices to provide remote monitoring of a person’s mobility or activities of daily living (ADLs) [19]. ADLs can be remotely monitored using motion detection devices installed in a person’s residence or a wearable device, such as a smart watch, that detects, records, and transmits movement activity. Another form of ADL monitoring is medication adherence monitored remotely via automated pillboxes. Automated pillboxes are used to organize medications, provide reminders to take medications, and provide information to clinicians via telehealth regarding medication use [20].

*DSSs* are electronic (computerized) systems which evaluate data collected via remote monitoring and transform the data into useful information regarding the patient’s health and wellness [21]. The DSS makes clinical or behavioral recommendations based on an evaluation of the monitored data. An example of a recommendation is a reminder to the patient to take his/her medication if an automated pillbox senses the person has not taken their medication that day. If the medication is still not taken after some delay, the DSS can notify the health care providers or health coaching system. The DSS can also initiate an emergency notification to 911 if certain threshold values of monitored parameters are exceeded.

Health coaching systems are defined as “patient-centered processes that are based upon behavior change theory” and include goal setting, education, encouragement, and feedback on health-related behaviors [14]. Disease management, by contrast, focuses on the specific disease(s) instead of the patient’s behavior [22]. Health coaching programs provide health-related information, recommendations, or encouragement to the patient on a routine or as-needed basis to help drive behavior changes [21]. Forms of health coaching include encouragement, feedback, health care suggestions, periodic health tips, or short educational presentations based on an analysis of the patient’s health status and monitored data. An example of a coaching message is sleep management advice if the patient is not sleeping well. The health coaching system can be manual (human health coach only), partially automated, or fully automated using artificial intelligence and machine learning to generate health coaching messages to the patient.

## Methods

**Figure 1:**
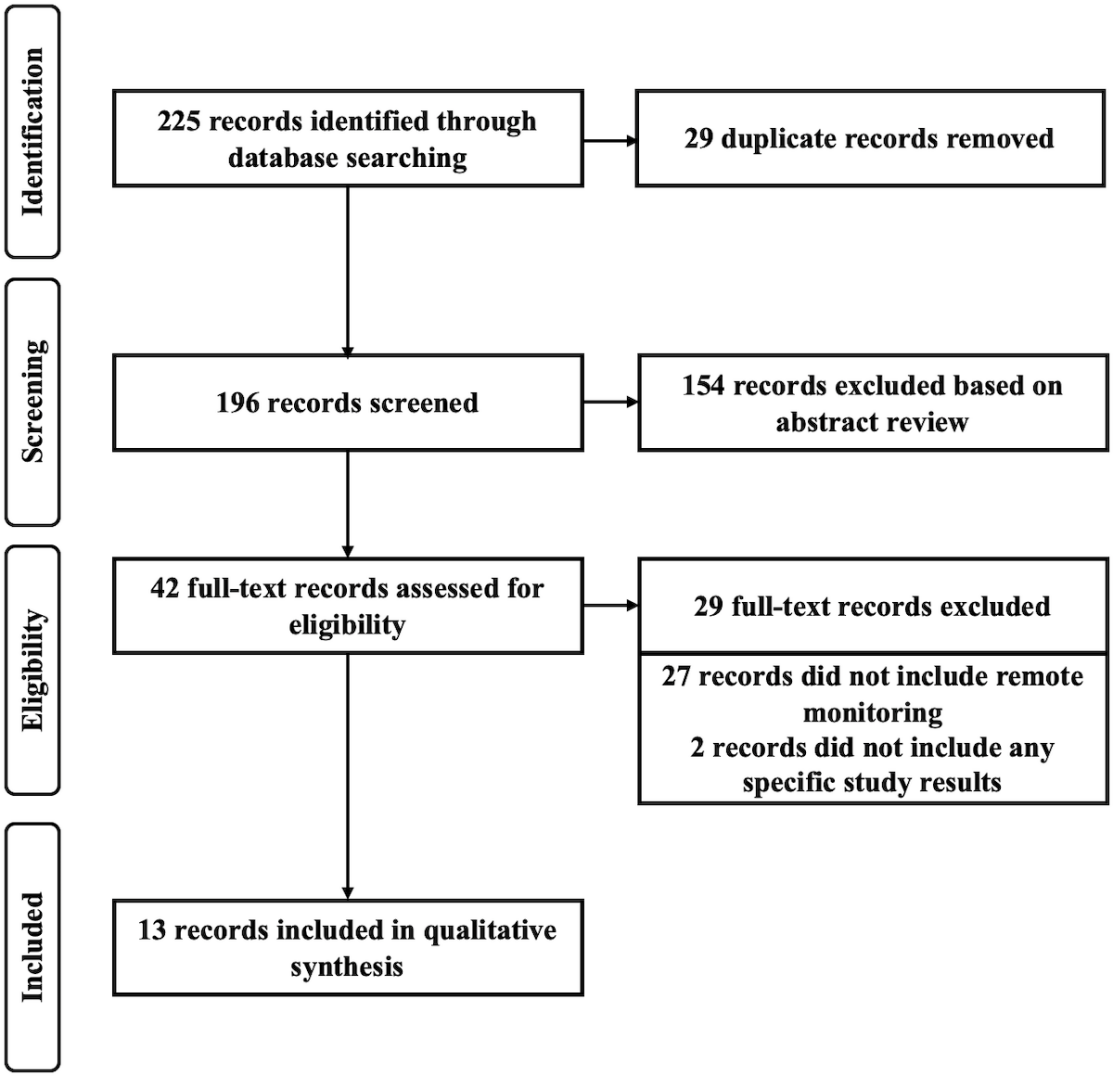
PRISMA (Preferred Reporting Items for Systematic Reviews and Meta-Analyses) flow chart.

A literature review was chosen for this study to identify the research conducted on the current state and effectiveness of health coaching combined with remote monitoring (RPM or RAM) and any knowledge gaps that warrant further research. This review was specifically focused on health coaching combined with telehealth to deliver health care with an emphasis on older adults. The Ovid MEDLINE and CINAHL databases were queried to first retrieve papers related to health or wellness coaching for populations that included older adults and to then narrow the results to those studies that included some form of remote monitoring. Given the rapid pace with which telehealth is advancing, results from 2010 or later were chosen for this search to focus on relatively current research. The full electronic search strategy was [(MH “Middle Aged”) OR (MH “Aged+”) OR AB (older adult* or elder* or aged) OR TI (older adult* or elder* or aged)] AND [AB ((health or wellness) n1 coaching) OR TI ((health or wellness) n1 coaching)]. The search criteria included articles published from January 2010 to September 5, 2019 (date of search) in English. Keywords included those related to older populations (aged, elder, and older adult) and coaching (health or wellness coaching). This combination of search terms retrieved 225 papers relevant to health coaching. The review of these papers was conducted in accordance with the Preferred Reporting Items for Systematic Reviews and Meta-Analyses (PRISMA) guidelines (Figure 1) [23]. After deleting duplicates, 196 papers were included for an abstract review and screening. These abstracts were reviewed for studies that discussed health coaching for populations that included older adults (aged 65 and older) combined with some form of remote monitoring. The abstract screening yielded 42 articles for full-text review, of which 13 articles were identified that met the eligibility criteria (health coaching, remote monitoring, and older adults). Studies were excluded from our review if older populations (aged 65 and over) were not included, if the study did not include remote monitoring (RPM or RAM), or if the study did not include some form of coaching intervention. Subsequent to the review, 2 additional studies were identified [24,25] which provided the results for the ACTIVATE Trial [26] included in the original search. The results of the literature review were charted based on the following criteria: description of the coaching intervention, type of remote monitoring, study type, size of the study population, length of the study, condition monitored, and the outcomes.

## Results

The results of the literature review are summarized in Table 1. All 13 studies were published between 2014 and 2019. Four studies were randomized controlled trials that ranged from 83 to 1437 participants [12,26-28]. One study was a quasi-experiment (nonrandomized cohort study) with 144 participants [29]. Six studies were pilot trials that ranged from 6 to 33 participants [21,30-34]. There was 1 qualitative interview of 10 health care workers [35] and 1 user acceptance study with 11 participants [36]. The main goal of each of these studies was to evaluate the effectiveness of health coaching. Our review focused on the effectiveness of health coaching (human coach versus automated coaching system) combined with remote monitoring technology (RAM and RPM) for older adults.

**Table 1:** Summary of results from the literature review.

Study	Coaching intervention	Type of remote monitoring	Study type	Sample size, n	Study duration	Condition monitored	Outcomes
[12]	Human coach and telephone calls	RPM^b^	Randomized control trial	1437	26 weeks	Chronic heart failure	Re-admissions (N^e^), mortality (N), and quality of life (Y^f^)
[27]	Human coach and telephone calls	RAM^c^ and RPM	Randomized control trial	595	1 year	Chronic heart failure, diabetes	Blood pressure (N), body weight (N), and quality of life (N)
[26]	Human coach and telephone calls	RAM	Randomized control trial	83	12 weeks	Cancer	Physical activity (Y) and sedentary behavior (Y)
[28]	Human coach, telephone calls, and mobile app	RAM and RPM	Randomized control trial	131	26 weeks	Diabetes	HbA_1c_^h^ level (N), body weight (Y), and quality of life (N)
[29]	Human coach, telephone calls, SMS text messages, online training, and social networking	RAM and RPM	Quasi-experiment	144	1 year	Diabetes	HbA_1c_ (Y) and body weight (Y)
[30]	Human coach, DSS^a^, telephone calls, and exercise videos	RAM and RPM	Pilot study	12	8 weeks	Chronic obstructive pulmonary disease	Program adherence (Y) and patient satisfaction (Y)
[31]	Human coach, telephone calls, and SMS text messages	RAM	Pilot study	24	4 weeks	Cancer	Physical activity (Y) and fatigue (Y)
[32]	Human coach, DSS, exercise videos, and SMS text messages	RAM	Pilot study	6	2-6 weeks	General health	Yes
[21]	Human coach, DSS, exercise videos, SMS text messages	RAM and RPM	Pilot study	33	Various	General health	Behavior change (I^g^)
[33]	Human coach, telephone calls, and mobile app	RAM and RPM	Pilot study	21	26 weeks	Diabetes	HbA_1c_ level (Y) and body weight (Y)
[34]	Automated coach, DSS, SMS text messages, and mobile app	RAM	Pilot study	27	26 weeks	Diabetes	HbA_1c_ level (Y) and activity level (Y)
[35]	Human coach and SMS text messages	RAM	Qualitative interview	10	N/A^d^	General health	Inconclusive
[36]	Human coach, telephone calls, and mobile app	RAM and RPM	User acceptance study	11	26 weeks	Diabetes	User acceptance (Y)

^a^DSS: decision support system.

^b^RPM: remote patient monitoring.

^c^RAM: remote activity monitoring

^d^N/A: not applicable.

^e^N: not effective.

^f^Y: effective.

^g^I: inconclusive.

^h^HbA_1c_: hemoglobin A_1c_.

The predominate type of health coaching was via a human coach (12/13 studies) [12,21,26-33,35,36], whereas an automated health coaching system was employed in only 1 study [34]. Human coaching included an initial training session [12,24-26,28,31,36], periodic training sessions [29], scheduled periodic contact with patients [12,24-27,29-31,33,35,36], or interventional contact based on remote monitoring results [12,21,28,32]. Four studies employed the use of a DSS to augment or assist the health coach [21,30,32,34]. The DSSs included software programs that generated trends and alerts for the health coach based on the remotely monitored data [30], artificial intelligence systems that evaluated the remotely monitored data and provided recommendations to the health coach [21,32], and a fully automated system that monitored physical activity and provided tailored feedback to the patient based on the monitored results [34]. Four studies employed the use of a mobile app for remote monitoring [28,33,34,36]. RAM was the most common type of telehealth technology employed (12 studies) [21,26-36] followed by RPM (8 studies) [12,21,27-30,33,36]. Communication with the patient was via telephone only (7 studies) [12,26-28,30,33,36], SMS text messages only (4 studies) [21,32,34,35], or telephone and SMS text messages (2 studies) [29,31]. Study durations ranged from 2 weeks to 1 year with 6 studies lasting 26 weeks or longer. The conditions monitored included diabetes (6 studies) [27-29,33,34,36] cancer (2 studies) [26,31], chronic heart failure (2 studies) [12,27], chronic obstructive pulmonary disease (1 study) [30], and overall general health (3 studies) [21,32,35].

Effectiveness outcomes assessed included hospital admissions/re-admissions, mortality, hemoglobin A_1c_ (HbA_1c_) level, body weight, blood pressure, physical activity level, fatigue, quality of life, and user acceptance of the coaching program and technology. Of the 13 studies reviewed, 10 found coaching supported by telehealth technology to be effective in at least one of the outcomes assessed in the studies [12,26,28-34,36]. As much as 5 of the 6 studies that monitored diabetes found health coaching plus remote monitoring to be effective particularly for physical activity level and body weight [28,29,33,34,36]. Neither of the 2 studies that monitored chronic heart failure found health coaching plus remote monitoring to be effective [12,27] except for improving one’s quality of life in one of the studies [12]. Both studies that monitored patients with cancer found health coaching plus remote monitoring to be effective at improving the patient’s physical activity level [26,31]. Only 1 [32] of the 3 studies that monitored general health [21,32,35] found health coaching plus remote monitoring to be effective. In summary, the results indicate that health coaching plus remote monitoring can be effective at improving a patient’s physical activity level, HbA_1c_ values, and in reducing body weight.

## Discussion

### Principal Findings

Health coaching that incorporates telehealth technologies has been implemented in older populations with mixed results. As much as 10 of the 13 studies reviewed found this method of health coaching to provide effective outcomes [12,26,28-34,36]. This literature review identified several gaps that warrant discussion or additional research.

### Human Versus Automated Coach

One of the more prominent findings identified in this review was the dependence on a human to provide health coaching and interaction with the patient. As much as 12 of the 13 studies reviewed included a human coach [12,21,26-33,35,36], and thus the outcomes were probably heavily reliant on a human in the process. Four of the studies did include health coaching systems that incorporated the use of a DSS [21,30,32,34]; however, only 1 study completely replaced the human coach with a DSS [34].

The health coaching system in the Yom-Tov et al’s pilot study [34] was fully automated in that neither the patient nor the health coach had to manually enter data or actions into the DSS or remote monitoring system after the patient’s activity goals were established. A smartphone app recorded the patient’s physical activity and transmitted the data to the DSS. A tailored daily feedback SMS text message was sent to each participant to encourage exercise. An algorithm determined the message to be sent based on whether the patient reached his/her activity goal the previous day. The study found that customizing or changing the daily message based on the actual physical activity performed was effective at getting the patient to increase daily activity whereas a constant daily reminder message was not effective. The use of a DSS to augment or replace human coaching indicates there is some movement toward augmenting the human coach with DSS technology. A benefit of using a DSS combined with remote monitoring is the ability to provide 24/7 continuous monitoring and intervention which may not be possible with a human coach. Although costs were not assessed in these studies, it is surmised that lessening the amount of direct human involvement in the coaching process should reduce overall cost. Additional studies should be performed with the focus of comparing the clinical and cost-effectiveness of the following 3 forms of health coaching: (1) human health coach only, (2) health coaching performed by a DSS only, and (3) a hybrid model of health coaching by a human coach augmented by a DSS.

### Telephone Versus Electronic Media Communications

Another finding identified in this review was the heavy reliance on the use of a telephone to communicate with patients. Nine of the studies used a telephone for delivering coaching with mixed effectiveness results (2 of these studies augmented telephone communications with SMS text messages) [12,26-31,33,36]. The other 4 studies used DSS messages, SMS text messages, or video messages in lieu of telephone calls, also with mixed effectiveness results [21,32,34,35]. These results indicate that coaching effectiveness may not be dependent on the method of communication with the patient. Additional studies should be performed to evaluate the effectiveness and acceptance of using electronic media to communicate with the patient instead of live telephone calls.

### Use of Smartphone Apps

Four studies included the use of a smartphone app as part of the integrated telehealth solution [28,33,34,36] with positive results for 3 of these studies [33,34,36]. Only one of these studies specifically evaluated the acceptance of smartphone app technology by the patients [36]. A recent qualitative study interviewed 12 community-dwelling older adults (aged 65-78) and found that older adults were, in general, satisfied with using technology to help monitor and manage their health on a daily basis (albeit amid some fears that technology would replace human contact) [37]. Thus, there appears to be an opportunity to expand the use of technology, such as smartphone apps, as part of a telehealth system for older adults.

### Coachability of Patients

Although not explicitly evaluated in the studies, it is probable that the results of these studies were dependent on the willingness of the patient to accept health coaching. Some of the studies evaluated the willingness of the patient to accept health coaching as part of the inclusion criteria while other studies only included patients who expressed an interest in the study. Thus, it can be assumed that most of the studies were biased toward those patients who are coachable. An opportunity exists to explore the effectiveness of health coaching using telehealth technology for patients who are not coachable.

### Limitations

This literature review was focused on studies that included older adults (aged 65 and older) in the population assessed. Studies that excluded older adults were not included in our review, so the results should not be extrapolated to general populations. Most of the coaching interventions reviewed in this study included a human coach who provided feedback to participants via telephone calls. This type of coaching depends on the effort of the human coach to provide an adequate type of coaching to the participant which may or may not include all aspects of a robust coaching program (goal setting, education, encouragement, and feedback on health-related behaviors). In addition, the studies reviewed did not attempt to assess the capability of a human coach versus an automated health coaching system to effect behavior change. Additional research is needed to make this assessment. There was only 1 fully automated coaching intervention study found in our review, so no conclusion can be drawn regarding the effectiveness of automated health coaching interventions. Additional research is needed in the area of automated health coaching. The search criteria for this review focused first on health and wellness coaching that was then further filtered on remote monitoring as an element of the coaching. Several other studies of telehealth might have included coaching but not as a focus of the study.

### Conclusions

Four inter-related issues face the health care industry: (1) the increasing numbers and percentage of older adults, (2) chronic disease management among this older population, (3) the desire of older adults to age in place, and (4) the cost of health care for older adults. Health coaching combined with telehealth technology has been shown to provide effective outcomes in 10 of 13 studies reviewed. Four studies included the use of a DSS to augment or replace the health coach with positive results. However, insufficient evidence of automated health coaching was found in our review to draw a conclusion regarding the efficacy of automated coaching. Although not assessed in these studies, the inclusion of automation in the health coaching process has the potential to reduce overall health care costs for older adults. The benefits of health coaching combined with telehealth are evident and should be further explored.

### Future Directions

One of the more prominent findings identified in this review was the dependence on a human to provide health coaching and interaction with the patient. Thus, the outcomes were probably heavily reliant on a human in the process. Future studies need to assess the capability of automated coaching systems versus human coaches to affect health behavior changes. Another prominent finding was the use of live telephone calls to provide coaching to the patient. Future studies should be performed to evaluate the effectiveness and acceptance of using electronic media to communicate with the patient. The studies reviewed did not specifically evaluate coachability or the willingness of the patient to accept health coaching. An opportunity exists to explore the effectiveness of health coaching using telehealth technology for patients who are not coachable. This discrepancy should be investigated by including quality of life measures in future studies of coaching systems. As sensors for RPM and RAM become more advanced and affordable, much more data will be available to monitor and evaluate. With advances in big data analytics, DSSs will be better informed and able to identify interventions when necessary. Based on the results of this review, additional studies should be conducted of the expanded use of health coaching and DSSs as part of the health care solution for older adults. In addition, cost-effectiveness of health coaching combined with telehealth needs to be assessed against human-only health coaching methods. The results of these studies would inform the future direction of health coaching.

## Abbreviations

ADL: activity of daily living
DSS: decision support system
PRISMA: Preferred Reporting Items for Systematic Reviews and Meta-Analyses
RAM: remote activity monitoring
RPM: remote patient monitoring
